# Molecular dynamics and functional characterization of I37R-CFTR lasso mutation provide insights into channel gating activity

**DOI:** 10.1016/j.isci.2021.103710

**Published:** 2021-12-31

**Authors:** Sharon L. Wong, Nikhil T. Awatade, Miro A. Astore, Katelin M. Allan, Michael J. Carnell, Iveta Slapetova, Po-chia Chen, Alexander Capraro, Laura K. Fawcett, Renee M. Whan, Renate Griffith, Chee Y. Ooi, Serdar Kuyucak, Adam Jaffe, Shafagh A. Waters

**Affiliations:** 1 School of Women's and Children's Health, Faculty of Medicine and Health, UNSW Sydney, Sydney, Australia; 2 Molecular and Integrative Cystic Fibrosis Research Centre (miCF_RC), UNSW Sydney, Sydney, Australia; 3 School of Physics, University of Sydney, Sydney, Australia; 4 Katharina Gaus Light Microscopy Facility, Mark Wainwright Analytical Centre, UNSW Sydney, Sydney, Australia; 5 Department of Respiratory Medicine, Sydney Children's Hospital, Randwick, Australia; 6 School of Chemistry, UNSW Sydney, Sydney, Australia; 7 Department of Gastroenterology, Sydney Children's Hospital, Randwick, Australia

**Keywords:** Pharmacology, Biochemistry, Structural biology

## Abstract

Characterization of I37R, a mutation located in the lasso motif of the CFTR chloride channel, was conducted by theratyping several CFTR modulators from both potentiator and corrector classes. Intestinal current measurements in rectal biopsies, forskolin-induced swelling (FIS) in intestinal organoids, and short circuit current measurements in organoid-derived monolayers from an individual with I37R/F508del CFTR genotype demonstrated that the I37R-CFTR results in a residual function defect amenable to treatment with potentiators and type III, but not type I, correctors. Molecular dynamics of I37R using an extended model of the phosphorylated, ATP-bound human CFTR identified an altered lasso motif conformation which results in an unfavorable strengthening of the interactions between the lasso motif, the regulatory (R) domain, and the transmembrane domain 2 (TMD2). Structural and functional characterization of the I37R-*CFTR* mutation increases understanding of CFTR channel regulation and provides a potential pathway to expand drug access to CF patients with ultra-rare genotypes.

## Highlights

• I37R-CFTR localizes to the cell surface and results in a residual function defect
• I37R-CFTR breaks a conserved salt bridge perturbing the lasso motif
• I37R-CFTR strengthens lasso interaction with the R domain, impacting channel gating
• I37R-CFTR is responsive to potentiators (GLPG1837) and class III correctors (VX-445)

## Graphical abstract

**Figure:**
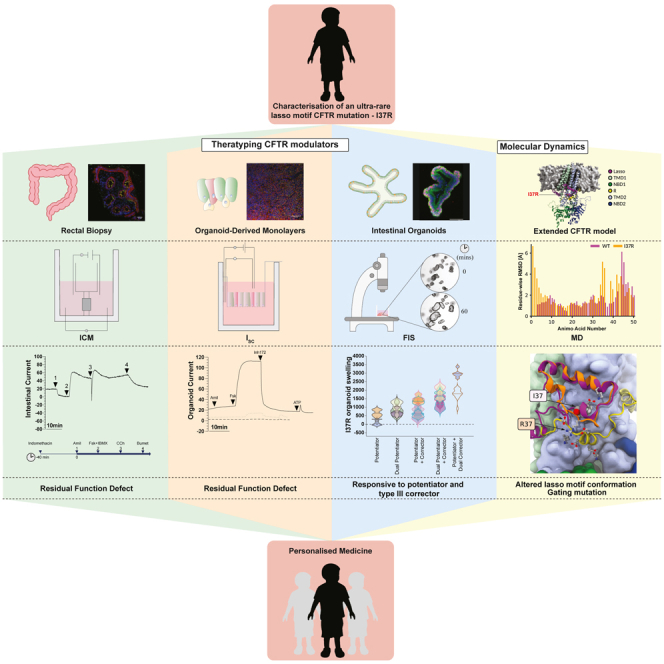

## Introduction

Cystic fibrosis (CF) is a life-limiting genetic disease resulting from mutations in the CF transmembrane conductance regulator (*CFTR*) gene (Ratjen et al., 2015). CFTR—the only member of the ABC transporter family known to be an ion channel—consists of two transmembrane domains (TMD1 and TMD2) which form an anion-selective pore, two highly conserved nucleotide-binding domains (NBD1 and NBD2) with ATP-binding pockets and a newly described N-terminal lasso motif (Hwang and Kirk, 2013; Zhang and Chen, 2016). In addition, CFTR has a unique, disordered regulatory (R) domain which contains protein kinase A (PKA) phosphorylation sites. For the CFTR channel to open and close (gate), cAMP-dependent PKA phosphorylation of the R domain first activates the CFTR (Gadsby and Nairn, 1994). Then, ATP-binding induces the dimerization of the two NBDs which opens the channel pore and ATP hydrolysis closes the pore.

The lasso motif (amino acids (aa) M1-L69), which is partially embedded in the bilayer and interacts with the R domain, was recently resolved following advancements in cryo-electron microscopy (cryo-EM) of the CFTR structure (Liu et al., 2017; Zhang et al., 2018). The first 40 amino acids of the lasso motif, which include lasso helix 1 (Lh1, aa V11–R29), form a circular “noose” structure (Hoffmann et al., 2018). The noose structure wraps around the transmembrane helices (TM2, TM6 of TMD1 and TM10, TM11 of TMD2) and is held in place by hydrophobic interactions with L15, F16, F17, T20, L24, and Y28. The C-terminal end of the lasso, which includes the lasso helix 2 (Lh2, aa A46–L61), is tucked under the elbow helix (aa I70–R75) (Hoffmann et al., 2018). Variable disease severity and heterogeneous clinical presentation have been reported for the 78 *CFTR* variants identified so far in the lasso motif (CFTR1 and CFTR2 databases, Table S1). Evidently, the lasso motif has a multifunctional role in CFTR regulation with variants impacting folding, gating, and stability of the CFTR protein (Fu et al., 2001; Gené et al., 2008; Jurkuvenaite et al., 2006; Sabusap et al., 2021; Thelin et al., 2007).

CFTR modulators, small molecules which directly target CFTR dysfunction, are now available to certain individuals with CF. Currently, two classes are approved; (1) potentiators, which open the channel pore such as ivacaftor (VX-770) and (2) correctors, which assist CFTR protein folding and delivery to the cell membrane. Type I correctors (lumacaftor/VX-809, tezacaftor/VX-661) stabilize the NBD1-TMD1 and/or NBD1-TMD2 interface by binding directly to TMD1 (Loo et al., 2013; Ren et al., 2013) or NBD1 which improves the interaction between NBD1 and the intracellular loops (Hudson et al., 2017; Loo and Clarke, 2017). Type II correctors (C4) stabilize NBD2 and its interface with other CFTR domains while type III correctors (elexacaftor/VX-445) directly stabilize NBD1 (Okiyoneda et al., 2013). Combination therapies of corrector(s) and a potentiator (Orkambi®, Symdeko/Symkevi®, Trikafta/Kaftrio®) have been approved for CF individuals with F508del, the most common *CFTR* mutation, as well as several specific residual function mutations. Most recently, Trikafta/Kaftrio has been approved for patients with a single F508del mutation in combination with a minimal function mutation, broadening the population of patients with CF eligible for treatment with CFTR modulator therapy.

Mounting evidence has shown that *in vitro* functional studies in patient-derived cell models successfully predict clinical benefit of available CFTR modulators for individuals bearing ultra-rare mutations (Berkers et al., 2019; McCarthy et al., 2018; Ramalho et al., 2021). In individuals with CF, adult stem cells are usually collected by taking either airway brushings or rectal biopsies. Single Lgr5^+^ stem cells, derived from crypts within a patient's intestinal epithelium, can be expanded in culture medium and differentiated into organized multicellular structures complete with the donor patient's genetic mutation(s), thus representing the individual patient (Sato et al., 2009). Stem cell models can be used for personalized drug screening to theratype and characterize rare CFTR mutations (Awatade et al., 2018; Berkers et al., 2019; Pollard and Pollard, 2018). Determining the functional response of rare, uncharacterized CFTR mutations to modulator agents with known CFTR correction mechanisms enables characterization of CFTR structural defects and enhances our understanding of CFTR function.

I37R-*CFTR* is a novel missense mutation in the lasso motif, detected in an Australian male child diagnosed through newborn screening with elevated immunoreactive trypsinogen, raised sweat chloride (>60 mmol/L), and CFTR Sanger sequencing identifying c.1521-1523del (F508del) and c.110C > T (I37R) mutations (Table S2). We used functional studies and molecular dynamics (MD) simulations to characterize the functional and structural defects of I37R-CFTR. CFTR function was assessed using intestinal current measurements (ICM) in rectal biopsies, forskolin-induced swelling (FIS) assays in intestinal organoids, and short circuit current measurements (I_sc_) in I37R/F508del organoid-derived monolayers, respectively. The potentiators VX-770 (approved), GLPG1837 (phase II clinical trials), and genistein (a natural food component with potentiator activity (Dey et al., 2016)) were tested as monotherapies, dual potentiator therapies, or in combination with correctors (VX-809, VX-661, and VX-445). We compared this to our laboratory reference intestinal organoids. For MD simulations, we modeled and examined the structural defect of the I37R mutation on an extended cryo-EM structure of ATP-bound, phosphorylated human CFTR (PDB ID code 6MSM) (Zhang et al., 2018).

## Results

### I37R-CFTR baseline activity in patient-derived rectal biopsies and intestinal organoids

**Figure 1:**
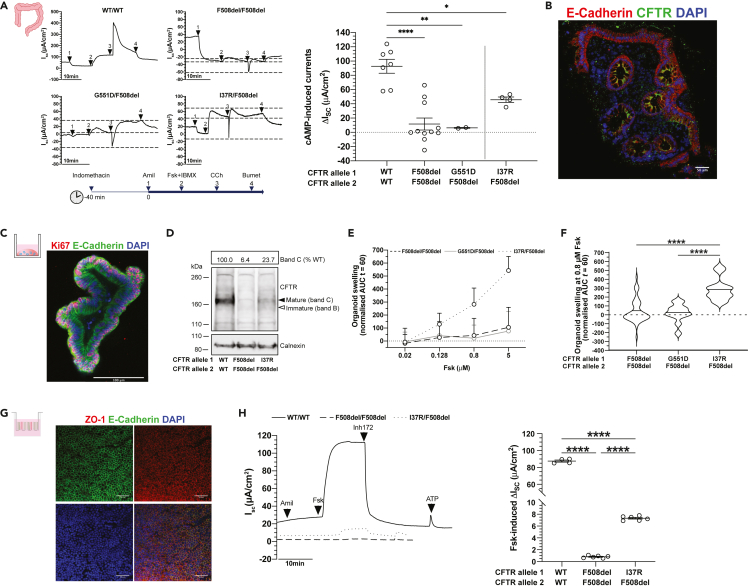
Characterization of I37R-CFTR residual function in rectal biopsies and intestinal organoids (A) Representative Ussing chamber recordings of intestinal current measurements (ICM) in rectal biopsies from WT-CFTR control participants and participants with CF. Dot plots of cAMP-induced current (ΔIsc-Fsk + IBMX) in participants with WT/WT (n = 2), F508del/F508del (n = 3), G551D/F508del (n = 1), and I37R/F508del (n = 1) CFTR genotypes. Experiments were performed in the presence of 10 μM indomethacin. Arrows indicate the addition of compounds: 100 μM apical amiloride (1. Amil), apical and basal addition of 10 μM forskolin +100 μM IBMX cocktail (2.Fsk + IBMX), 100 μM basal carbachol (3.CCh), and 100 μM basal bumetanide (4.Bumet). The Isc at the time CCh was added (middle horizontal dotted line), and the maximum (top dotted lines) and minimum (bottom dotted lines) Isc induced are indicated. Each dot represents an individual replicate. (B) Immunofluorescence staining of CFTR (green), e-cadherin (red), and DAPI (blue) in a rectal biopsy derived from an I37R/F508del participant. 63x/1.4 oil immersion objective. Scale bar = 50 μm. (C) Immunofluorescence staining of e-cadherin (green), Ki67 (red), and DAPI (blue) in intestinal organoids derived from an I37R/F508del participant. 20x/0.75 dry objective. Scale bar = 100 μm. (D)Western blot in WT/WT, F508del/F508del, and I37R/F508del intestinal organoids. CFTR maturation was calculated by measuring the level of mature mutant CFTR (Band C) as a percentage of mature CFTR from WT organoids (% normal CFTR). All data were normalized to the calnexin loading control. B and C represents the mature, complex-glycosylated CFTR. B and B represents the immature, core-glycosylated CFTR. See Figure S9 for uncropped Western blot images. (E and F) Forskolin-induced swelling (FIS) assay in organoids from participants with F508del/F508del (n = 5), G551D/F508del (n = 2), and I37R/F508del (n = 1) CFTR genotypes. Organoids were stimulated with forskolin (fsk) concentrations ranging from 0.02 to 5 μM.(E) FIS expressed as the means ± standard deviation (SD) of the area under the curve (AUC) calculated from t = 0 (baseline) to t = 60.(F) FIS of organoids at 0.8μM fsk at baseline represent residual CFTR function. Data represented as violin plots with mean to show distribution. (G) Immunofluorescence staining of e-cadherin (green), ZO-1 (red), and DAPI (blue) in organoid-derived monolayers from a CF participant. 20x/0.75 dry objective. Scale bars = 50 μm. (H) Representative Ussing chamber recordings of short circuit current in organoid-derived monolayers from a WT-CFTR control participant and participants with CF. Dot plots of fsk-induced current (ΔIsc-Fsk) in participants with WT/WT (n = 1), F508del/F508del (n = 1), and I37R/F508del (n = 1) CFTR genotypes. Experiments were performed in the presence of 10 μM indomethacin. Arrows indicate the addition of compounds: 100 μM apical amiloride, 5 μM basal fsk, 30 μM apical CFTR inhibitor CFTRinh-172, and 100 μM apical ATP. Each dot represents an individual replicate. Data in (A) and (H) represented as mean ± standard error of the mean (SEM). One-way analysis of variance (ANOVA) was used to determine statistical differences. ∗p < 0.05, ∗∗p < 0.01, ∗∗∗∗p < 0.0001.

Intestinal current measurements (ICM) were performed on I37R/F508del and reference CF (F508del/F508del, G551D/F508del) and non-CF (wild-type: WT/WT) rectal biopsies using a standard protocol (Clancy et al., 2013; Graeber et al., 2015) (Figure 1A). Following stimulation with a forskolin (fsk) and IBMX cocktail, rectal biopsies from the I37R/F508del CF participant elicited cAMP-dependent currents of 45.8 ± 3.8 μA/cm^2^—an appreciable 50% of WT-CFTR activity (p < 0.05; Figure 1A, Table S3). This response was at least 4-fold higher than those of the reference CF biopsies, although statistical significance was not reached.

Co-activation with carbachol (CCh) resulted in a biphasic response in the I37R/F508del biopsies, characteristic of residual CFTR chloride channel function in the CF colon (Graeber et al., 2015; Veeze et al., 1994). The initial negative I_sc_ peak indicates apical potassium secretion reached 9.4 ± 2.5 μA/cm^2^. Following this, the CCh-induced positive I_sc_ indicates the increase of apical chloride secretion reached 15.78 ± 2.07 μA/cm^2^. This biphasic response was similarly observed in the G551D/F508del biopsies (25.77 ± 2.16 μA/cm^2^) but was diminished in the F508del/F508del biopsies (−2.28 ± 1.65 μA/cm^2^). These findings are in accordance with the localization of CFTR protein at the plasma membrane (mature complex-glycosylated CFTR) of the I37R/F508del rectal biopsies, as demonstrated by immunofluorescence staining (green; Figure 1B).

Next, CFTR protein expression and maturation was assessed in I37R/F508del, reference F508del/F508del, and WT/WT organoids using Western blot (Figures 1C and 1D). The expression of complex-glycosylated C band in I37R/F508del organoids was 23.7% that of the WT/WT organoids, considerably higher than the 6.4% detected from F508del/F508del organoids (Figure 1D). CFTR activity was then evaluated in I37R/F508del and CF reference intestinal organoids using a fsk-induced swelling (FIS) assay at four fsk concentrations between 0.02 and 5 μM (Figure 1E). FIS of I37R/F508del intestinal organoids at 0.8 μM fsk—the optimal concentration for baseline assessment of CFTR activity (Dekkers et al., 2016a)—was 282.9 ± 36.0 (Figures 1E and 1F). This exceeded the baseline FIS of the reference intestinal organoids by at least 7-fold (F508del/F508del: AUC = 42.8 ± 19.4; G551D/F508del: AUC = 21.3 ± 29.4).

The morphological difference between WT (pre-swollen) and CF organoids (Cuyx et al., 2021), means comparing CFTR activity between CF and healthy CFTR function by FIS assay cannot be achieved (Dekkers et al., 2016a; van Mourik et al., 2019). In order to compare I37R/F508del to wild-type CFTR activity, organoid-derived monolayers were created (Figure 1G) and CFTR ion transport was performed (Zomer-van Ommen et al., 2018). Fsk-stimulated CFTR-dependent currents were 9-fold higher in I37R/F508del monolayers than those of reference F508del/F508del monolayers (7.3 ± 0.2 vs 0.8 ± 0.1 μA/cm^2^; p < 0.0001), but 12-fold lower than WT/WT monolayers (87.5 ± 1.3 μA/cm^2^; p < 0.0001) (Figure 1H). This is consistent with the FIS assay results demonstrating high baseline CFTR activity in I37R/F508del intestinal organoids.

### I37R-CFTR functional response to CFTR modulator monotherapy in intestinal organoids

**Figure 2:**
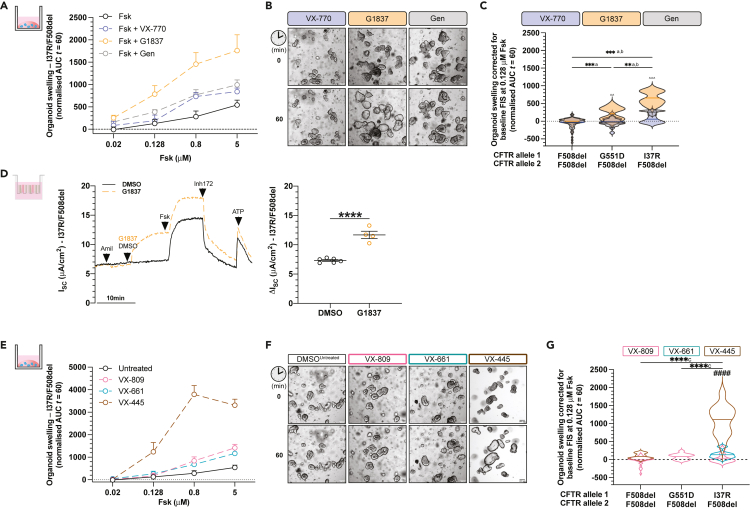
Characterization of I37R-CFTR functional response to corrector or potentiator monotherapy in intestinal organoids Forskolin-induced swelling (FIS) assay in organoids from participants with F508del/F508del (n = 5), G551D/F508del (n = 2), and I37R/F508del (n = 1) CFTR genotypes. Organoids were incubated overnight with 0.03% DMSO (untreated) or 3 μM VX-809 or 3 μM VX-661 or 3 μM VX-445. After 24 h, organoids were stimulated with fsk concentrations ranging from 0.02 to 5 μM, either alone or in combination with potentiator monotherapy (3 μM VX-770 or 3 μM G1837 or 50 μM Gen). (A) FIS of I37R/F508del organoids stimulated with VX-770, GLPG1837 (G1837), or genistein (Gen) monotherapy, expressed as the means ± standard deviation (SD) of the area under the curve (AUC) calculated from t = 0 (baseline) to t = 60 min. (B) Representative brightfield images of I37R/F508del organoids at baseline (t = 0) and after 1 h of stimulation (t = 60) at 0.128 μM fsk. Scale bars = 100 μm. (C) FIS of organoids at 0.128uM fsk following stimulation with VX-770, GLPG1837 (G1837), or genistein (Gen) monotherapy. Data corrected for baseline FIS and represented as violin plots with mean to show distribution. (D) Representative Ussing chamber recordings of short circuit current in I37R/F508del organoid-derived monolayers. Dot plots of total currents stimulated by DMSO or G1837 plus fsk. Experiments were performed in the presence of 10 μM indomethacin. Arrows indicate the addition of compounds: 100 μM apical amiloride, apical addition of either vehicle control 0.01% DMSO or 10 μM G1837, 5 μM basal fsk, 30 μM apical CFTR inhibitor CFTRinh-172, and 100 μM apical ATP. Each dot represents an individual replicate. Data represented as mean ± standard error of the mean (SEM). (E) FIS of I37R/F508del organoids pre-incubated with corrector (VX-809 or VX-661 or VX-445) for 24 h, expressed as the means ± standard deviation (SD) of the area under the curve (AUC) calculated from t = 0 (baseline) to t = 60 min. (F) Representative brightfield images of I37R/F508del organoids at baseline (t = 0) and after 1 h of stimulation (t = 60) at 0.128 μM fsk. Scale bars = 100 μm. (G) FIS of organoids at 0.128uM fsk following incubation with corrector (VX-809 or VX-661 or VX-445) for 24 h. Data corrected for baseline FIS and represented as violin plots with mean to show distribution. One-way analysis of variance (ANOVA) was used to determine statistical differences except in (D) where unpaired t test was used. ∗∗p < 0.01, ∗∗∗p < 0.001, and ∗∗∗∗p < 0.0001. aP for G1837, bP for Gen and cP for VX-445 of I37R/F508del, ˆP for G1837 vs VX-770, or Gen and #P for VX-445 vs VX-809 or VX-661.

We investigated the functional response of I37R/F508del organoids to single potentiators—VX-770, GLPG1837 (G1837), and genistein (Gen). Treatment with VX-770 minimally increased FIS of I37R/F508del organoids by AUC of 59.7 above baseline at 0.128 μM fsk (Figures 2A–2C and S1)—the optimal concentration for *in vitro* assessment of CFTR modulator response to predict clinical effect (Dekkers et al., 2016a). G1837 and Gen both significantly increased FIS, albeit with different efficacies (655.8 and 256.8, respectively; Figures 2A–2C and S1). None of the potentiator treatments increased FIS in F508del/F508del organoids, indicating no improvement in CFTR activity in response to potentiator therapy (Figure 2C). Only G1837 significantly increased FIS in the G551D/F508del organoids (210.4 ± 57.5; p < 0.01). In comparison to G551D/F508del organoids, G1837 was 3-fold more efficacious in the I37R/F508del organoids (p < 0.0001).

Because G1837 demonstrated the greatest restoration of CFTR activity in I37R/F508del organoids, we evaluated G1837 treatment of I37R/F508del organoid-derived monolayers. G1837 led to a significant 1.5-fold increase in fsk-stimulated currents (ΔI_sc_: 4.4 μA/cm^2^; p < 0.0001) (Figure 2D). This is consistent with the FIS of I37R/F508del organoids, indicating that I37R-CFTR responds to potentiator agents.

Given the I37R/F508del high residual CFTR activity and its localization at the epithelial cell surface, we hypothesized that the I37R-*CFTR* mutation has minimal impact on CFTR protein folding or maturation. Treatment of I37R/F508del organoids with type I corrector agents (VX-809 or VX-661) did not significantly increase FIS above baseline (Figures 2E–2G and S1). In contrast, treatment of I37R/F508del organoids with a type III corrector agent (VX-445) significantly increased FIS by AUC of 1112.5 above baseline, greater than those in the F508del/F508del organoids (42.5). VX-445 has been shown to act as both a corrector and potentiator for certain *CFTR* mutations (Laselva et al., 2021; Shaughnessy et al., 2021; Veit et al., 2021a). Acute treatment of I37R/F508del organoids with VX-445 did not improve potentiation of CFTR (Figure S1). This supports the observation that VX-445-stimulated rescue of CFTR in I37R/F508del organoids acts by a correction mechanism improving I37R mild folding and processing defects. I37R-CFTR functional response to CFTR modulator co-therapies in intestinal organoids.

**Figure 3:**
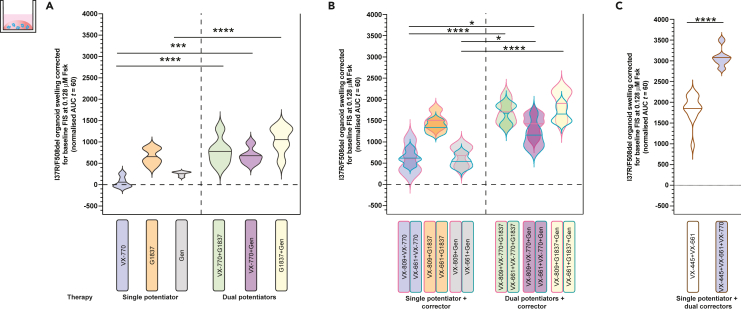
Characterization of I37R-CFTR functional response to dual potentiator or corrector therapy or corrector(s)-potentiator(s) co-therapy in intestinal organoids Forskolin-induced swelling (FIS) assay in organoids from participants with F508del/F508del (n = 5), G551D/F508del (n = 2), and I37R/F508del (n = 1) CFTR genotypes. (A and B) Organoids were incubated overnight with 0.03% DMSO (untreated) or 3 μM VX-809 or 3 μM VX-661 or 3 μM VX-445 + 18 μM VX-661. After 24 h, organoids were stimulated with fsk ranging in concentration from 0.02 to 5 μM, either alone or in combination with a single potentiator (3 μM VX-770 or 3 μM G1837 or 50 μM Gen) or dual potentiators (VX-770 + G1837 or VX-770 + Gen or G1837 + Gen). FIS of organoids at 0.128uM fsk stimulated with VX-770, GLPG1837 (G1837), or genistein (Gen) or their combinations, following (A) 24 h pre-incubation with DMSO (untreated) or (B) corrector (VX-809 or VX-661), respectively. (C) FIS of organoids at 0.128uM fsk stimulated without or with VX-770, following 24 h pre-incubation with dual correctors (VX-445+VX-661). Data corrected for baseline FIS and represented as violin plots with mean to show distribution. One-way analysis of variance (ANOVA) was used to determine statistical differences except in (C) where unpaired t test was used. ∗p < 0.05, ∗∗∗p < 0.001, ∗∗∗∗p < 0.0001

Combination treatments of CFTR modulators are used to treat patients bearing *CFTR* mutations with multiple functional defects such as F508del and patients who are heterozygous for CFTR mutations. We investigated the effect of combinations of potentiators. Dual potentiator combinations increased FIS of I37R/F508del organoids to a greater extent than the respective single potentiators (Figure 3A) and had a synergistic effect, where the FIS was greater than the sum of the respective single potentiators (Table S4). Despite G1837 + Gen having greater efficacy than the other dual potentiator combinations, the magnitude of response was not statistically different between the different combinations of dual potentiators (Figure 3A).

Co-therapy with a corrector (VX-809 or VX-661) and dual potentiators significantly (p < 0.01) increased FIS of I37R/F508del organoids compared to co-therapy of a corrector with VX-770 or Gen, but not G1837 (Figure 3B). VX-809/G1837 + Gen co-therapy had the greatest efficacy, increasing FIS 1904.0 above baseline. In contrast, corrector/VX-770 + Gen co-therapy had the least efficacy. This trend was consistent with that of the dual potentiators synergistic effect.

Dual correctors (VX-445+VX-661) increased FIS in I37R/F508del organoids by AUC of 1856.6 above baseline, which corresponds with the level of rescue achieved by the most effective corrector/dual potentiator co-therapy (VX-809/G1837 + Gen). The triple combination therapy with dual correctors and a potentiator further increased FIS in I37R/F508del organoids by AUC of 3101.6 above baseline. It is therefore the most effective modulator combination tested in this study.

### I37R-CFTR perturbs the noose structure of the lasso motif

**Figure 4:**
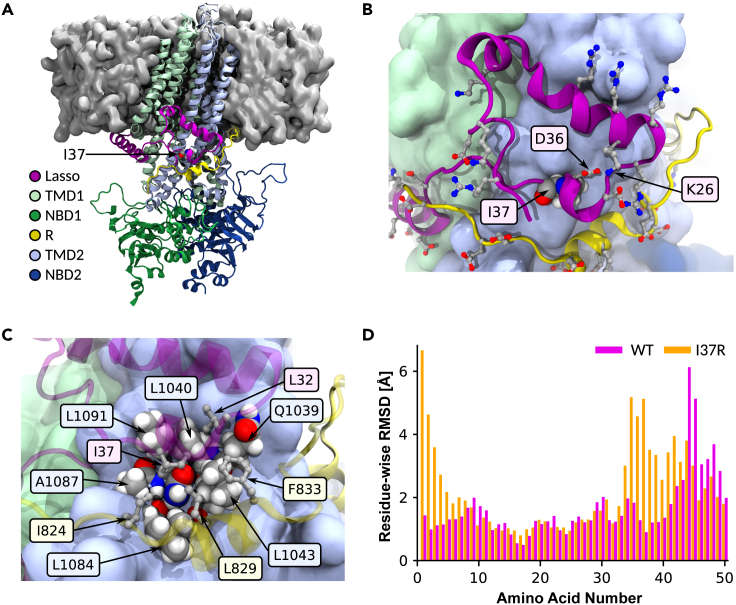
Placement of I37R within the lasso motif and the resulting changes to its conformation (A) Ribbon structure of human CFTR, partially embedded within the plasma membrane (gray surface). I37 rendered as spheres. TMD: transmembrane domain; NBD: nucleotide-binding domain; R: regulatory domain (R domain). (B) K26-D36 salt bridge stabilizing the noose structure of the lasso motif. Charged amino acids depicted as balls and sticks. I37 rendered as spheres, and color-coded by element (gray: C; white: H; red: O; blue: N). (C) I37 positioned within a hydrophobic pocket formed by amino acids from the lasso motif, TMD2, and poorly resolved R domain. Relevant amino acids labeled and depicted as spheres in TMD2, and as balls and sticks in lasso motif and R domain. (D) Residue-wise root-mean-square deviation (RMSD) to the C-alpha atoms of the WT-CFTR 6MSM model, measuring the conformational change of the WT (pink) and I37R mutant (orange) lasso after 2 μs simulations. Values are means sampled over the last μs of simulations.

We next characterized the structural defect of I37R-CFTR using MD simulations. The primary structure of the lasso motif (M1-L69) is conserved across 230 vertebrate species (Figure S2, Table S5). The lasso motif formed a noose structure that rested against TMD2 (Figure 4A). Amino acids V12-R29 were embedded in the plasma membrane while the rest of the lasso motif resided in the cytosol. The noose structure was maintained by a salt bridge formed between K26 and D36 (Figure 4B). I37 was positioned in the center of this noose, within a hydrophobic pocket formed by amino acids from the lasso, TMD2, and the poorly resolved R domain in the cytosol (Figure 4C).

Mutation of the evolutionarily conserved, non-polar and uncharged isoleucine (I) of I37 to a positively charged arginine (R) introduced an unstable lone charge into the hydrophobic pocket within the lasso motif noose. We hypothesized that this likely results in the rotation of the R37 side chain out of the hydrophobic pocket, and possible coordination with negative charges in the nearby R domain.

To identify a reasonable conformation of the mutant lasso motif, the WT 6MSM model was mutated to R37 and three 2 μs simulations were performed at physiological temperature (310 K). The R37 side chain rotated out of the hydrophobic pocket in only one of the three simulations. The difference between the root-mean-square deviation (RMSD) of the noose structure of I37R-CFTR compared to the WT was on average 2.8 Å at the amino acids M1-L6, and 1.8 Å at L34-S50 (Figure 4D). To confirm this observation, repeat simulations were performed at 350 K (40° above physiological temperature), a temperature shown to accelerate the potential conformational transitions of proteins (Beckerman, 2015). In these higher temperature simulations, the root-mean-square fluctuation (RMSF) of the region around amino acid 37 doubled in two out of three simulations, compared to WT-CFTR at 310K (Figure S3). This confirmed the destabilization of the lasso motif by I37R-CFTR. All WT-CFTR domains and the surrounding bilayer remained stable at the elevated temperature (Figures S4 and S5).

### I37R mutation strengthens lasso motif interaction with the R domain

**Figure 5:**
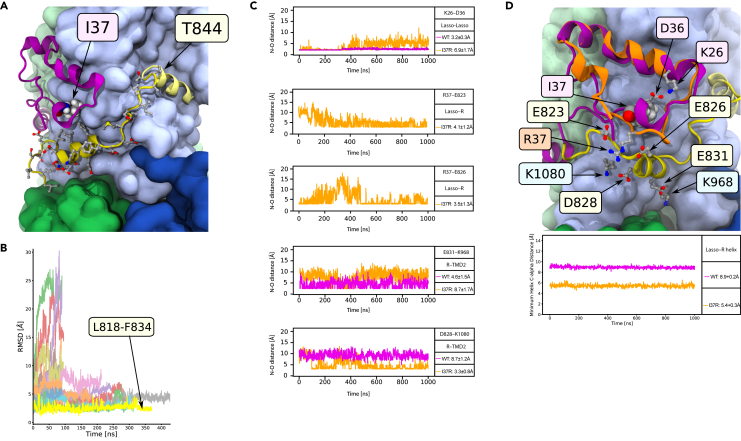
I37R interacts with a previously unresolved section of the R domain (A) The reconstructed R domain amino acids (yellow), depicting the assignment of L818-F834 to the 17 amino acids with only the backbone resolved in the 6MSM structure, and the linking residues to T845 in TMD2. Lasso motif in purple. Side chains depicted as balls and sticks. (B) The stabilities of all 24 modeled R domain assignments, quantified by RMSD to the 6MSM structure. The most stable alignment of the 17 unidentified amino acids, L818-F834, is highlighted yellow. The full list of tested assignments is shown in Supplementary material 9. (C) The minimum N-O distance between newly formed and disrupted salt bridges in the I37R mutant. Distance less than 4 Å indicates direct contact. Values are means ± standard deviations (SD), sampled over the last 500 ns of simulations. (D) Conformational changes in the I37R mutant (orange) compared to WT (purple) lasso motif, which brings it closer to the R domain (yellow). Minimum C-alpha atom distance between amino acid 37 and the R domain helix (E826-F834) in I37R and WT.

In the 6MSM structure, the R domain is largely unresolved with two exceptions: the first (Q637) and last (T845) amino acids that adjoin neighboring domains, and the backbone atoms of a 17 amino acid segment. This latter segment consists of an eight amino acid disordered coil followed by a nine amino acid alpha-helix (Zhang et al., 2018). The alpha-helix was separated by approximately 10 Å (1 nm) minimum C-alpha distance to I37 in the lasso motif. This suggested a likely interaction between this segment of the R domain and I37, which necessitated partial modeling of the R domain (Figure 5A).

Modeling of these 17 unidentified amino acids was performed by creating 24 different *in silico* models of this segment based on the 6MSM structure. In each model, a unique 17 amino acid sequence was determined with a sliding window of one amino acid, starting backwards from amino acid T842 due to the alpha-helix’s 20 Å proximity to T845. The 17 amino acids were then connected to T845 with the missing linking amino acids. The structural stability of all 24 modeled segments was tested by performing up to 300 ns simulations for each model and comparing the backbone RMSD measurements against 6MSM (Figures 5B and S6). The model with the lowest RMSD (3 Å) and thus the highest stability was attained when L818-F834 was assigned to the unidentified 17 amino acids, of which the alpha-helix maps to E826-F834 (Figures 5B and S6). This assignment was corroborated by NMR measurements of the isolated R domain in solution, where the same segment retained partial helicity (Baker et al., 2007). Predictions of the structure of human CFTR by Alphafold2 also aligned with this assignment of primary structure to the unidentified amino acids (Figure S7) (Jumper et al., 2021). Several favorable interactions between this R domain model and other parts of the CFTR protein further supported this assignment (Figures 4C and 5D). Two hydrophobic amino acids (L829 and F833) contributed to the hydrophobic pocket that stabilized the lasso motif around I37. The negatively charged E831 formed a salt bridge with positively charged K968 in TMD2. Together, these interactions secured the R domain alpha-helix into position throughout an extended 2 μs simulation, resulting in a smaller minimum C-alpha distance to the lasso motif of 8.9 ± 0.2 Å compared to the 10 Å in the 6MSM cryo-EM structure.

The reoriented R in position 37 in the I37R mutant protein, which pointed out of the hydrophobic pocket, rearranged the salt bridge network supporting the lasso motif by breaking the evolutionarily conserved salt bridge K26–D36. Two new salt bridges were formed, one with the negatively charged E823 and another with E826 of the R domain (Figure 5C). Furthermore, the E831–K968 salt bridge between the R and TMD2 domains in the WT was exchanged for a D828–K1080 salt bridge in I37R-CFTR (Figure 5C). The backbone motions required to accommodate these new charge interactions also perturbed parts of the lasso motif (Figure S3) and R domain. The lasso N-terminus shifted its position towards the R domain and reduced the minimum C-alpha distance between them by 3.5 Å (Figure 5D). The overall result was a tighter coupling between the lasso and the R domain which is anticipated to inhibit the R domain movements required for channel gating.

## Discussion

We have described the functional and structural defects of I37R, a novel CF-causing mutation in the segment of the CFTR lasso motif which interacts with the R domain. These were compared to reference *CFTR* mutations which have known functional defects, either a CFTR folding/maturation (F508del/F508del) or a gating (G551D/F508del) defect. First, ICM performed in I37R/F508del rectal biopsies identified I37R confers high residual activity (50% of WT-CFTR activity). High baseline CFTR activity was similarly observed in FIS of I37R/F508del intestinal organoids and I_sc_ measurements in organoid-derived monolayers. Given we and others showed that F508del is a severe mutation which contributes little functional CFTR (Van Goor et al., 2011), this suggests that I37R mutation produces CFTR protein which localizes to the epithelial cell surface. These observations are consistent with the patient's mild CF clinical phenotypes (pancreatic sufficient with faecal elastase>500 μg/g, FEV_1_ z-score -0.11, 99% predicted).

We also characterized the response of I37R-CFTR to modulators (potentiators and correctors) in I37R/F508del intestinal organoids and organoid-derived monolayers. I37R was responsive to potentiators which improve CFTR gating function and a newly approved corrector (VX-445). Among the three potentiator agents tested, the response to VX-770 was minimal. The reason for the lack of efficacy of VX-770 is not known, because molecular modeling studies propose that VX-770 shares the same mechanism of action and binding sites with G1837 (Liu et al., 2019; Yeh et al., 2019). Both VX-770 and G1837 are proposed to potentiate CFTR by increasing channel open probability (Po) through stabilization of the open-pore conformation, independent of NBD dimerization and ATP hydrolysis which normally controls channel gating (Van Goor et al., 2009; Yeh et al., 2017). However, the differing potentiator efficacies are not a new observation. G1837 was previously shown to be more potent and effective than VX-770 in human bronchial epithelial cells from a G551D/F508del and a R334W/F508del CF participant (Gees et al., 2018; Van der Plas et al., 2018). Similar observations were reported in heterologous HEK293 cells expressing Class III (G551D, G178R, and S549N) and Class IV (R117H) CFTR mutants (Gees et al., 2018; Van der Plas et al., 2018). We conclude that perhaps G1837 has additional binding sites or actions distinct from VX-770, which in the case of I37R-CFTR, results in significant potentiation of the CFTR channel.

We further showed that dual potentiator combinations exerted synergistic restoration of CFTR activity in I37R/F508del organoids. This synergistic restoration is not exclusive to I37R-CFTR, because similar findings have been reported for other *CFTR* mutations responsive to potentiators (Dekkers et al., 2016b; Phuan et al., 2018, 2019; Veit et al., 2019). Synergism is commonly achieved when potentiators have distinct binding sites and mechanisms of actions. One potentiator could induce allosteric interactions that favor the activity of the other potentiator (Nussinov and Tsai, 2013). The potentiator synergy observed in our dual potentiator combinations supports our hypothesis that G1837 may have additional binding sites or mechanisms of action to VX-770. While VX-770 has been shown to provide clinical benefit to patients with responsive mutations (Berkers et al., 2020; McKone et al., 2014; Volkova et al., 2020), it does not restore the Po of gating defect mutants (G551D-CFTR) to full WT-CFTR activity (Van Goor et al., 2009). This opens the possibility that using another potentiator with a different mechanism of action could complement VX-770 activity and increase CFTR activity beyond that of VX-770 monotherapy. While VX-770 and G1837 act independently of NBD dimerization and ATP hydrolysis (Van Goor et al., 2009; Yeh et al., 2017), genistein promotes ATP-dependent gating of CFTR by binding to the NBD1/2 interface and inhibiting ATP hydrolysis (Sohma et al., 2013). Genistein has been demonstrated to increase VX-770-potentiated CFTR activity in intestinal organoids, even when VX-770 was used at near-saturating concentrations (Dekkers et al., 2016b). Our observations reiterate and expand on these findings to suggest that potentiators with different mechanisms of action could provide synergistic restoration of CFTR activity to responsive *CFTR* mutations compared to potentiator monotherapy.

Chronic treatment with type III corrector VX-445 rescued CFTR activity in I37R/F508del organoids, while neither type I correctors (VX-809 or VX-661) rescued activity. This response is attributed to the I37R and not the F508del mutation in the I37R/F508del organoids, because VX-445 did not restore CFTR activity in F508del/F508del organoids. While VX-445 has been shown to have partial potentiator activity (Laselva et al., 2021; Shaughnessy et al., 2021; Veit et al., 2021a), VX-445 did not potentiate CFTR activity in I37R/F508del organoids when administered acutely. This is the first study to interrogate the potentiator action of VX-445 in intestinal organoids; however, previous studies have been performed in donor-derived bronchial and nasal epithelial cells and immortalized cell lines. The higher correction efficacy of VX-445 when compared with VX-809/VX-661 has previously been shown, although this is likely to be dependent on the CFTR variant (Keating et al., 2018; Veit et al., 2020, 2021b). For instance, direct binding of VX-445 to NBD1 to stabilize and prevent the domain unfolding may make it more effective in correcting CFTR mutations that impact NBD1 function (such as F508del located in NBD1).

The lack of I37R-CFTR correction by VX-809 or VX-661 could be attributed to the dependency of these modulators binding to and stabilizing the TMD1. TMD1 function is modulated by interaction with lasso helix 2 (Lh2, aa A46–L61) as deletion of Lh2 from the WT CFTR was shown to completely abrogate VX-809-mediated CFTR maturation (Sabusap et al., 2021). MD studies showed that VX-809 occupancy at the TMD1 binding site causes the Lh2 to move, such that the network of salt bridges in Lh2 holds TMD1 (CL1) and TMD2 (CL4) in the correct orientation (Baatallah et al., 2021; Okiyoneda et al., 2013). This then allows for allosteric coupling between NBD1 and TMD1 or 2, which is important for cooperative domain folding of CFTR. In support of this, mutation of critical amino acids at the binding pocket of VX-809 on CFTR, or those involved in the architecture of this site, were shown to diminish the sensitivity to VX-809 correction. L53V and F87L mutations, which are located in the vicinity of the VX-809 binding site in the TMD1, were shown to prevent VX-809 correction in F508del HEK283 cells (Baatallah et al., 2021). Considering the above and because I37 is only a few amino acids away from the Lh2, it is plausible that the local conformational changes associated with the I37R mutation which we have identified in our study (Figure 4D) may disrupt the allosteric coupling between NBD1 and TMD1 or 2, preventing correction with type I correctors.

*CFTR* missense mutations in the lasso motif are not well characterized. This is because most of these mutations are rare, with an allele frequency of less than 0.01% in the CF population (Table S1). The only characterized missense mutations in the region of the lasso motif where I37 resides—between Lh1 (amino acid 19–29) and Lh2 (amino acid 46–61)—are R31C and R31L (CFTR2, 2021; Jurkuvenaite et al., 2006). Experimental studies in heterologous COS-7 cells showed both mutations cause a mild processing defect and accelerated CFTR internalization. Individuals heterozygous for these *CFTR* mutations are reported to have a mild disease phenotype with pancreatic sufficiency (Jurkuvenaite et al., 2006). One individual with the R31C/F508del *CFTR* genotype was reported to have a normal sweat chloride level (25 mmol/L) and nasal potential difference (Werlin et al., 2015). CFTR2 classifies R31C as a non-CF disease causing mutation. Notably, mild disease phenotypes (mild pulmonary symptoms, pancreatic sufficiency) are reported for several other lasso motif missense mutations including P5L, E56K, and P67L (Table S1), as was found for the I37R/F508del participant in this study. This suggests that perhaps lasso motif mutations do not significantly impact the overall CFTR structure and function given its short length (69 of 1480 amino acids, 4.7%). It is also plausible that the role of the lasso motif could be compensated for by other CFTR domains.

To better understand the functional defect of I37R-CFTR, we used MD simulations to model the structural features of I37R and how they are altered relative to WT-CFTR. The amino acids 34–39 were shown to interact with the R domain in the phosphorylated, ATP-bound CFTR structure (Zhang et al., 2018). This interaction was absent in the closed conformation of CFTR (Zhang and Chen, 2016), suggesting that the short region of amino acids 34–39 interacts with the R domain to regulate CFTR channel gating. We found that the disruption of the evolutionarily conserved K26-D36 salt bridge in I37R-CFTR brings the lasso motif closer to the R domain. We also found that the I37R side chain rotates out of its hydrophobic pocket to form interactions with negatively charged E823 and E826 on the R domain. We speculate that R37 clamps the lasso motif to the R domain, preventing the dynamic movement of the two domains necessary for a normal CFTR opening and closing cycle, thus causing a gating defect. This supports our functional observations, wherein I37R-CFTR demonstrated significant responsiveness to potentiator agents which are known to increase channel opening time. Furthermore, in the I37R-CFTR model, conformational changes in the lasso motif were also evident but were limited to short regions (M1-L6, L34-S50), indicating that the overall architecture of the CFTR protein remains largely intact. Additionally, our simulations did not show any change to the pore architecture of CFTR (Figure S8).

The simulated structure in this work is of CFTR in its active state (Zhang et al., 2018). Because of this, we believe the pathogenic interactions discovered in this study have a significant contribution to the deleterious effects of the I37R mutation. However, the enhanced lasso motif-R domain interactions should be interpreted in the context of the μs timescales reachable by unbiased simulations. The lasso domain is known to exhibit conformational flexibility during both folding and functional stages of CFTR (Kleizen et al., 2021), which take place on timescales longer than is currently feasible to study in atomistic simulations. Therefore, there may be pathogenic interactions in I37R-CFTR in addition to the ones captured by the simulation of this particular CFTR structure.

The I37R/F508del participant in this study will only meet the Therapeutic Goods Administration (Australia) requirements for treatment with Trikafta/Kaftrio triple combination therapy once he turns 12 years old given the single copy of the F508del mutation. He is not eligible for single potentiator therapy or corrector/potentiator combinations of lumacaftor/ivacaftor or tezacaftor/ivacaftor. This emphasizes the importance of characterizing the structural and functional defects of ultra-rare *CFTR* mutations together with the assessment of *in vitro* response to modulator drugs in patient-derived cell models to build the case for access to treatment with available modulators through precision medicine health technology assessment pathways. Furthermore, when multiple CFTR modulators are available to patients with CF, determining the best modulator for patients with a rare mutation not investigated in a clinical trial may be supported using *in vitro* personalized cell models.

### Limitations of the study

Organoids often lack specialized cell types and fail to recapitulate the complexity of native organs (Clevers, 2016). For example, mesenchymal, endothelial, and microbiome are absent from intestinal organoids. Integration of such features remains technically challenging and their absence may impact drug response. Another important drawback of organoid systems is the heterogeneity in their size when seeded for FIS assay. As the size of organoids increases, diffusion-dependent drug supply becomes less efficient. This may in turn impact the accuracy of outcome of drug assay. Reducing this variability will be essential to fully capitalize on the potential of organoids in drug screening. Another limitation of the organoid systems is the variability in the magnitude of FIS response in intestinal organoids across different CF laboratories. This is due to the dependence of organoids on media that is developed in-house with many locally produced media factors (Dekkers et al., 2016a; Ramalho et al., 2021). This limitation can be resolved by the creation of reference donor organoids which are made available and used internationally between CF laboratories.

## STAR★Methods

### Key resources table

**Table:** 

REAGENT or RESOURCE	SOURCE	IDENTIFIER
**Antibodies**		

Mouse monoclonal anti-CFTR (CF3)	Abcam	Cat#ab2784; RRID:AB_303297
Rabbit polyclonal anti-Ki67	Abcam	Cat#ab15580; RRID:AB_443209
Rabbit monoclonal anti-E-cadherin (24E10)	Cell Signalling	Cat#3195; RRID:AB_2291471
Rabbit monoclonal anti-calnexin (C5C9)	Cell Signalling	Cat#2679; RRID:AB_2228381
Mouse monoclonal anti-E-cadherin (HECD-1)	Life Technologies	Cat#13-1700; RRID:AB_211510
Rabbit polyclonal anti-ZO-1	Life Technologies	Cat#61-7300; RRID:AB_11207989
Mouse anti-CFTR	University of North Carolina and Cystic Fibrosis Foundation	Cat#596

**Biological samples**		

Human rectal biopsies	Molecular and Integrative Cystic Fibrosis (miCF) Research Centre Biobank	https://wch.med.unsw.edu.au/micf-research-centre

**Chemicals, peptides, and recombinant proteins**		

Matrigel, growth factor reduced, phenol-free	Corning	Cat#356231
Indomethacin	Sigma-Aldrich	Cat#I7378
Amiloride	Sigma-Aldrich	Cat#A7410
Forskolin	Sigma-Aldrich	Cat#F6886
IBMX (3-Isobutyl-1-methylxanthine)	Sigma-Aldrich	Cat#I5879
Carbachol	Sigma-Aldrich	Cat#C4382
Bumetanide	Sigma-Aldrich	Cat#B3023
CFTRInh-172	Sigma-Aldrich	Cat#C2992
ATP	Sigma-Aldrich	Cat#A2383
Calcein AM	Thermo Fisher	Cat#C3100MP
VX-445	Selleckchem	Cat#S8851
VX-661	Selleckchem	Cat#S7059
VX-770	Selleckchem	Cat#S1144
VX-809	Selleckchem	Cat#S1565
GLPG1837 (ABBV-974)	Selleckchem	Cat#S8698

**Deposited data**		

Molecular structure of the ATP-bound, phosphorylated human CFTR	(Zhang et al., 2018)	PDB: 6MSM
Molecular dynamics equilibrated structure of cryo-EM model of CFTR with added R domain fragment	This publication	https://doi.org/10.5281/zenodo.5615645
Human CFTR canonical isoform (primary structure)	UniProtKB	Identifier: P13569-1

**Experimental models: Organisms/strains**		

Human intestinal organoids	Molecular and Integrative Cystic Fibrosis (miCF) Research Centre Biobank	https://wch.med.unsw.edu.au/micf-research-centre
Human intestinal organoid-derived monolayers	Molecular and Integrative Cystic Fibrosis (miCF) Research Centre Biobank	https://wch.med.unsw.edu.au/micf-research-centre

**Software and algorithms**		

Image J version 1.53c	National Institutes of Health, Bethesda, MD	https://imagej.nih.gov/ij/index.html
Prism version 9.2.0	GraphPad Software, San Diego, CA	https://www.graphpad.com/
Modeller 9.19	(Sali and Blundell, 1993)	https://salilab.org/modeller/
GROMACS version 2019.3	(Abraham et al., 2015)	https://manual.gromacs.org/
VMD version 1.9.3	(Humphrey et al., 1996)	https://www.ks.uiuc.edu/Development/Download/download.cgi?PackageName=VMD
MDAnalysis version 1.0.0	(Michaud-Agrawal et al., 2011)	https://www.mdanalysis.org/
FATSLiM version 0.2.2	(Buchoux, 2017)	https://fatslim.github.io/
Molecular dynamics and analysis workflow	This publication	https://doi.org/10.5281/zenodo.5615645

**Other**		

VCC MC8 Ussing chambers	Physiologic Instruments, San Diego, CA	–
Zeiss Axio Observer Z.1 inverted microscope	Carl Zeiss, Jena, Germany	–
Leica TCS SP8 DLS confocal microscope	Leica Microsystems, Wetzlar, Germany	–

### Resource availability

#### Lead contact

Further information and requests for resources and reagents should be directed to and will be fulfilled by the Lead Contact, Dr Shafagh Waters (shafagh.waters@unsw.edu.au).

#### Materials availability

This study did not generate new unique reagent.

#### Data and code availability

• MD simulations data of an equilibrated model of CFTR with the missing R domain fragment has been deposited at Zenodo and is publicly available as of the date of publication. DOI is listed in the key resources table. All other data reported in this paper will be shared by the lead contact upon request.
• All original code for MD studies has been deposited at Zenodo and is publicly available as of the date of publication. DOI is listed in the key resources table. The code for FIS assay will be shared by the lead contact upon request.
• Any additional information required to reanalyze the data reported in this paper is available from the lead contact upon request.

### Experimental model and subject details

#### Participants biospecimen collection

Rectal biopsies were collected from CF participants with I37R/F508del (n=1), F508del/F508del (n=6) and G551D/F508del (n=2) *CFTR* genotypes, as well as WT-*CFTR* control participants (n=3) (Supplementary material 2). Samples were taken during investigative or surveillance endoscopy. This study was approved by the Sydney Children’s Hospital Ethics Review Board (HREC/16/SCHN/120). Written informed consent was obtained from all participating subjects or their legal guardians.

#### Intestinal organoid culture from rectal biopsies

Organoid cultures were established from crypts isolated from four to six rectal biopsies (Dekkers et al., 2016a). Rectal biopsies were washed with cold PBS, and incubated with 10 mM EDTA (Life Technologies 15575-020) in PBS at 4°C for 120 min on a tube rotator. EDTA solution was discarded and crypts were dislodged by vigorous pipetting of biopsies in cold PBS. Isolated crypts were seeded in 70% matrigel (Growth factor reduced, phenol-free; Corning 356231) in 24-well plates at a density of ∼10 – 30 crypts in 3x10 μl matrigel droplets per well. Media change was performed every second day and organoids were passaged 1:3 after 7 – 10 days of culture. Organoid culture media is made up of advanced DMEM/F-12 media, 10 mM HEPES, 2 mM L-glutamine, 1x B-27 Supplement, 1.25 mM N-acetylcysteine, 50 ng/ml hEGF, 50% Wnt3a-conditioned media, 300 ng/ml hRspo-1, 100 ng/ml hNoggin, 10 mM nicotinamide, 500 nM A83-01, 10 μM SB202190, 1x penicillin-streptomycin, 50 μg/ml gentamicin, 50 μg/ml vancomycin, 250 ng/ml fungizone and 100 μg/ml primocin (Wong et al., 2021).

#### Organoid-derived monolayers cultures

Monolayers cultures were created from 7-day-old intestinal organoids which were dissociated into single cells using TrypLE Express Enzyme (Life Technologies 12605-010) at 37°C for 2x2 min (Zomer-van Ommen et al., 2018). Mechanical disruption was performed after each incubation period. Cells were then seeded on collagen I-coated (Advanced Biomatrix 5005) Transwell 6.5mm, 0.4 μm pore polyester membrane inserts (Sigma CLS3470) at a density of 250,000 cells per insert. Cells were cultured with organoid culture media supplemented with 10 μM Y-27632 for 48 h. Media change (without Y-27632) was performed every second day for 7 days.

### Method details

#### Intestinal current measurement

Superficial rectal mucosa samples (2 – 4 per donor) were freshly obtained using biopsy forceps (CK Surgitech NBF53-11023230) and placed in cold RPMI1640 media (Sigma R5886) with 5% FBS. Intestinal current measurements were performed under voltage-clamp conditions using VCC MC8 Ussing chambers (Physiologic Instruments, San Diego, CA) (De Jonge et al., 2004; Derichs et al., 2010; Li et al., 2004). Biopsy tissues were bathed in Ringer solution containing (mM): 145 NaCl, 3.3 K_2_HPO_4_, 0.4 KH_2_PO_4_, 10 D-Glucose, 10 NaHCO_3_, 1.2 MgCl_2_ and 1.2 CaCl_2_. Ringer solutions were continuously gassed with 95% O_2_-5% CO_2_ and maintained at 37˚C. 10 μM indomethacin was added to both apical and basal chambers, and tissues were stabilised for 40 min. Tissues were then treated with pharmacological compounds (in order): 100 μM amiloride (apical) to inhibit epithelial sodium channel (ENaC)-mediated Na^+^ flux, 10 μM forskolin + 100 μM IBMX cocktail (apical and basal) to induce cAMP activation of CFTR, 100 μM carbachol (basal) to increase intracellular Ca^2+^ levels and activate basolateral Ca^2+^-dependent K^+^ channels and 100 μM bumetanide (basal) to inhibit basolateral Na^+^/K^+^/2Cl^-^ (NKCC) co-transporter.

#### Forskolin-induced swelling assay

Passage 3-15 organoids were seeded in 96-well plates, in 4 μl 70% matrigel droplet per well containing ∼25–30 organoids. The next day, organoids were incubated with 1.84 μM calcein green (Thermo Fisher Scientific C3100MP) for at least 30 min prior to addition of fsk at 0.02, 0.128, 0.8 or 5 μM concentrations, to determine cell viability. For CFTR potentiation, a single potentiator (3 μM VX-770 or 3 μM G1837 or 50 μM Gen) or dual potentiators (VX-770+G1837 or VX-770+Gen or G1837+Gen) was added together with fsk. Time-lapse images of organoid swelling were acquired at 10-min intervals for 60 min at 37°C using Zeiss Axio Observer Z.1 inverted microscope (Carl Zeiss, Jena, Germany), on an EC Plan-Neofluar 5x/0.16 M27 dry objective. Organoids were pre-incubated with 3 μM VX-809 or 3 μM VX-661 or 3 μM VX-445 or 3 μM VX-445+18 μM VX-661 for 24 h prior to FIS for CFTR correction where indicated. Three wells were used per condition and each participant’s FIS experiment was repeated 3 to 4 times.

#### Quantification of forskolin-induced swelling

Organoid swelling was quantified using a custom-built script. A segmentation strategy implemented using ImageJ/Fiji was performed on brightfield images. The raw image was processed with a gaussian blur (s=1.3) to reduce noise. After the directionality and magnitude of the local gradient was identified, pixels were classified as either ‘Background’, ‘Ridge’, ‘Valley’, ‘Rising’ or ‘Falling’ dependent on their neighbouring pixels along the previously calculated local directionality. Clean-up filters were applied that remove noise and small objects, such as ridges that only touched background pixels, and erosions to decrease rising and falling edges to better approximate object boundaries (‘Peaks’). A size exclusion was applied that would discriminate debris in the sample preparation from organoids of interest. This segmentation strategy was used to identify area covered by organoid at each time point. The total surface area of organoid at 10-min intervals over 60 min post-fsk stimulation were calculated and normalized against t=0 to render the relative amount of swelling from t=0. The area under the curve, AUC (calculated increase in organoid surface area from t=0 to t=60; baseline=100%) was then calculated using GraphPad Prism software.

#### Quantification of CFTR-mediated ion transport in organoid-derived monolayers

Short circuit current (I_sc_) measurements were performed under voltage-clamp conditions using VCC MC8 Ussing chambers (Physiologic Instruments, San Diego, CA). Cells were bathed in 20 mM HEPES buffered-Ringer solution containing (mM): 120 NaCl, 0.8 K_2_HPO_4_, 5 D-Glucose, 1.2 MgCl_2_ and 1.2 CaCl_2_. Ringer solutions were continuously gassed with 95% O_2_-5% CO_2_ and maintained at 37˚C. 10 μM indomethacin was added to both apical and basal chambers and cells were stabilised for 15 min. Cells were then treated with pharmacological compounds (in order): 100 μM amiloride (apical) to inhibit epithelial sodium channel (ENaC)-mediated Na^+^ flux, vehicle control 0.01% DMSO or 10 μM G1837 (apical) to potentiate cAMP-activated currents, 5 μM forskolin (basal) to induce cAMP activation of CFTR, 30 μM CFTR_inh_-172 (apical) to inhibit CFTR-specific currents and 100 μM ATP (apical) to activate calcium-activated chloride currents. I_sc_ in response to forskolin was considered as baseline activity (ΔI_sc-Fsk_) and I_sc_ in response to forskolin and potentiator (ΔI_sc-Fsk+Pot_) was used as the measure of modulator response.

#### Immunofluorescence

A rectal biopsy from a I37R/F508del participant was embedded in Tissue-Tek Optimal Cutting Temperature (OCT) compound (Sakura Finetek, CA) and snap frozen prior to storage at -80°C. The frozen biopsy was cut into 4 μm slice sections, and the sections were fixed in ice-cold methanol for 15 min. Intestinal organoids cultured from the I37R/F508del participant and organoid-derived monolayers cultured from a F508del/F508del participant were fixed in 4% paraformaldehyde and ice-cold methanol respectively for 15 min. Fixed samples were blocked using IF buffer (0.1% BSA, 0.2% Triton and 0.05% Tween 20 in PBS) with 10% normal goat serum (Sigma G9023) for 1 h at room temperature before incubation in primary antibodies overnight at 4°C. The biopsy section was stained with CFTR (1:50, Abcam ab2784) and E-cadherin (1:100, Cell Signalling 3195) antibodies. Intestinal organoids were stained with Ki67 (1:250, Abcam ab15580) and E-cadherin (1:250, Life Technologies 13-1700) antibodies. Organoid-derived monolayers were stained with ZO-1 (1:250, Life Technologies 61-7300) and E-cadherin (1:250, Life Technologies 13-1700) antibodies. On the following day, samples were washed with IF buffer 3 times, 5 min each and incubated with Alexa Fluor conjugated secondary antibodies (1:500, Life Technologies A-11029, A-21329) for 1 h at room temperature. Samples were mounted with Vectashield hardset antifade mounting medium containing DAPI (Vector Laboratories H-1500). Images were acquired using Leica TCS SP8 DLS confocal microscope (Leica Microsystems, Wetzlar, Germany), either on a 63x/1.4 or a 20x/0.75 objective. Images were processed using ImageJ (National Institutes of Health, Bethesda, MD).

#### Western blotting

Intestinal organoids were lysed with TNI lysis buffer (0.5% Igepal CA-630, 50 mM Tris pH 7.5, 250 mM NaCl, 1 mM EDTA) (Pankow et al., 2015) containing protease inhibitor cocktail (Roche 04693159001) on ice for 30 min. Lysates were then sonicated using the Bioruptor Pico (Diagenode, Liège, Belgium) at 4°C for 20 cycles of 30 sec on and 30 sec off. Lysates were spun down at 14,000 rpm at 4°C for 20 min and protein concentrations were determined using the BCA Protein Assay Kit (Thermo Fisher Scientific 23225). Lysates (100 μg per sample) were separated using NuPAGE 3 – 8% Tris-Acetate gels (Thermo Fisher Scientific EA0375BOX) at 100 V for 30 min, followed by 150 V until separation was complete. Proteins were transferred onto a nitrocellulose membrane using wet transfer at 20 V for 1 h at RT. The membrane was then incubated in 5% non-fat dry milk in phosphate-buffered saline containing 0.1% Tween (PBST) for 1 h at RT. CFTR bands were detected using anti-CFTR antibody 596 (1:500; University of North Carolina, Chapel Hill and Cystic Fibrosis Foundation) incubated at 4°C overnight. Protein bands were visualised using ECL Select detection reagent (Cytiva RPN2235) on the ImageQuant LAS 4000 (GE Healthcare, Chicago, IL). Calnexin was used as the loading control, detected using anti-calnexin antibody (1:1000; Cell Signalling Technology 2679). Protein band densitometry was performed using ImageJ (National Institutes of Health, Bethesda, MD). CFTR maturation in I37R/F508del and F508del/F508del organoids were estimated by measuring the level of mature mutant CFTR (band C) as a percentage of mature CFTR from WT organoids (% normal CFTR) (Van Goor et al., 2014).

#### *In silico* system composition

A 1-palmitoyl-2-oleoyl-sn-glycero-3-phosphocholine (POPC) bilayer was generated using the VMD membrane builder plugin (Humphrey et al., 1996) in which a model based on the phosphorylated human CFTR channel (PDB ID: 6MSM) was embedded (Zhang et al., 2018). The system was solvated with TIP3P water and neutralised with 0.15 M of potassium chloride ions (Mark and Nilsson, 2001). The WT-CFTR system included 236 POPC molecules, 128 potassium ions, 140 chloride ions and 44503 water molecules.

#### Extended 6MSM structure: modelling the unidentified section of the R domain

The 6MSM structure was extended in order to resolve a previously unassigned section in the R domain. The R domain is 227 residues long (F630-H856) and is largely disordered (Bozoky et al., 2013). In the 6MSM structure, the sidechains of 17 residues of this domain are labelled “UNKNOWN”, due to inadequate electron density in the region. The first 8 residues are unstructured while the next 9 residues form an alpha helix. The distance between the end of the helix and the first visible residue in TMD2 (T845) is 20 Å (Zhang et al., 2018). Using VMD’s autopsf plugin (Humphrey et al., 1996) we populated the side chains of the unknown section. Modeller 9.19 was then used to link the R domain to TMD2 at T845 (Sali and Blundell, 1993). 24 possible primary structure alignments of this region were simulated. The Root Mean Squared Deviation (RMSD) of the backbone alpha carbon atoms of the extended section with respect to the 6MSM structure was calculated over 300 ns of MD simulations. The most stable alignment was chosen from the lowest RMSD compared to the 6MSM structure. The most stable configureuration was capped with the neutral forms of the C and N termini and incorporated into our CFTR model. Four other missing loops namely residues 410-434, 890-899, 1174-1201, 1452-1480 were reconstructed using Modeller 9.19, based on visual analysis and the lowest discrete optimised protein energy (DOPE) score (Shen and Sali, 2006). The N and C termini of the CFTR model were capped with the physiological, charged termini.

#### Molecular dynamics simulation protocols

The 6MSM structure carries an engineered mutation to avoid the hydrolysis of the bound ATP, giving it a longer lifetime in the open conformation (E1371Q). This mutation was corrected to match the WT-CFTR sequence using the mutator plugin of VMD. The I37R missense mutation was constructed in the same way. GROMACS v2019.3 with the CHARMM36m forcefield was used for all MD simulations (Abraham et al., 2015; Huang et al., 2017). Minimisation via a steepest descent algorithm was performed until all forces were below 24 kcal/mol/Å. This was followed by relaxation simulations of all heavy atoms in the system starting with a restraint of 10 kcal/mol/Å^2^ and then halving this restraint every 200 ps in 15 iterations. Relaxation and production were run with 1 and 2 fs time steps, respectively. Relaxation was followed by 5 ns of equilibration. During relaxation, a Berendsen thermostat and barostat were applied, and for production a Nosé-Hoover and Parrinello-Rahman thermostat and barostat were applied respectively (Berendsen et al., 1984; Nosé and Klein, 1983; Parrinello and Rahman, 1981). To maintain the area per lipid (APL) properties of the POPC membrane at experimental values during production runs, pressure coupling was applied in the z-direction normal to the membrane bilayer while the x-y dimensions of the cubic simulation volume was fixed (Klauda et al., 2010). While semi-isotropic pressure coupling better replicates membrane environments (Pandit and Scott, 2008), this constant area approach was adopted to circumvent an issue with GROMACS 2019.3reb (https://gitlab.com/gromacs/gromacs/-/issues/2867). Production runs were extended up to 2 μs at 310 K with three replicates for all simple MD simulations. The last 1 μs of the longest simulations for each system were selected for further analysis. This was the longest time feasible to simulate with available computational resources. All RMSDs were calculated using the positions of alpha carbons with reference to the 6MSM experimental structure (Zhang et al., 2018). Analysis scripts were written in python using the MDAnalysis library (Gowers et al., 2016; Michaud-Agrawal et al., 2011). Bilayer thickness and area per lipid were calculated with the FATSLiM software package (Buchoux, 2017).

#### Mathematical formulae

Root Mean Square Deviation (RMSD)

$$RMSD\left(t\right)=\sqrt{\frac{1}{n}\sum {\left|x_{i}\left(t\right)-x_{i}^{ref}\right|}^{2}}$$

Root Mean Square Fluctuation (RMSF)

$$RMSF_{i}=\sqrt{{\left|x_{i}-x_{i}^{ref}\right|}^{2}}$$

Angular brackets indicate a time average.

### Quantification and statistical analysis

Data for Figures 1A, 1G and 2D are presented as dot plots with mean ± standard error of the mean (SEM). Data for Figures 1D, 2A and 2E are presented as line graphs with mean ± standard deviation (SD). Data in Figures 1E, 2C, 2G, 3A, and 3B are presented as violin plots with mean. One-way analysis of variance (ANOVA) or unpaired t-test was used to determine statistical differences as indicated. Statistical analysis was performed with GraphPad Prism software v9.0.1. A *p* value of less than 0.05 was considered to be statistically significant.

## Supplementalinformation

Document S1. Figures S1–S9 and Tables S2–S4

Table S1. List of lasso motif mutations, related to Figure 1

Table S5. CFTR accession number across different species, related to Figure 4A
