# Supplementary material for: Molecular dynamics and functional characterization of I37R-CFTR lasso mutation provide insights into channel gating activity
Source: iScience. 2021 Dec 31;25(1):103710. doi: 10.1016/j.isci.2021.103710 (PMC8761696; doi:10.1016/j.isci.2021.103710)
Supplement: Document S1. Figures S1–S9 and Tables S2–S4 [file mmc1.pdf]

iScience, Volume *25*

Supplemental information

Molecular dynamics and functional characterization of I37R-CFTR lasso mutation provide insights into channel gating activity

Sharon L. Wong, Nikhil T. Awatade, Miro A. Astore, Katelin M. Allan, Michael J. Carnell, Iveta Slapetova, Po-chia Chen, Alexander Capraro, Laura K. Fawcett, Renee M. Whan, Renate Griffith, Chee Y. Ooi, Serdar Kuyucak, Adam Jaffe, and Shafagh A. Waters

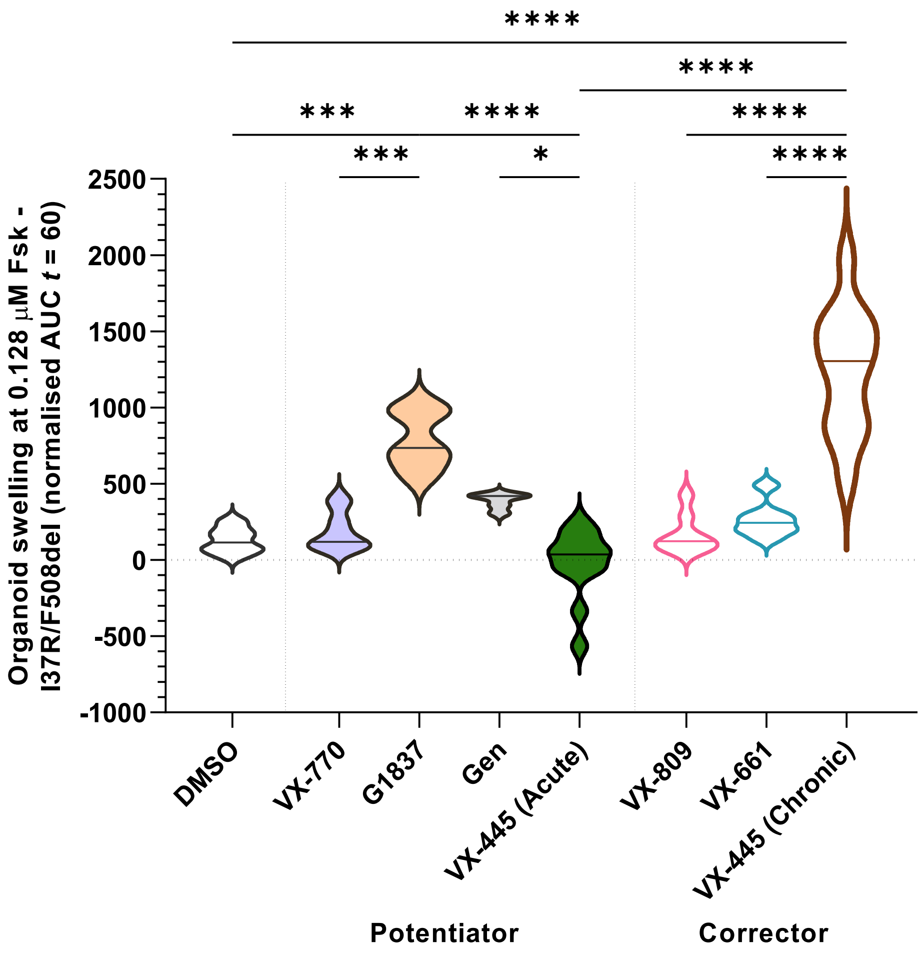

Figure S1. Forskolin-induced swelling (FIS) of I37R/F508del intestinal organoids in response to corrector and potentiator monotherapy at 0.128 µM forskolin without correction for baseline FIS, related to Fig 2C and 2G. One-way analysis of variance (ANOVA) was used to determine statistical differences. *P < 0.05, ***P < 0.001 and ****P < 0.0001.

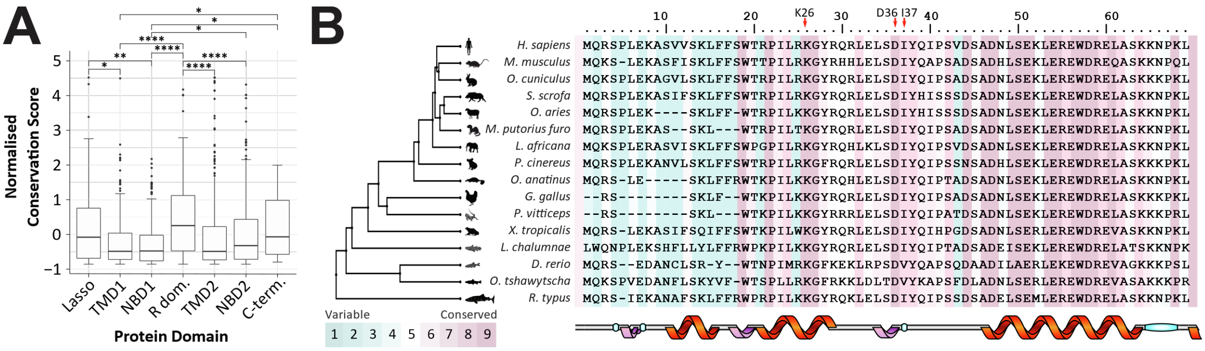

Figure S2. Sequence conservation of the lasso motif, related to Fig 4A. (A) Conservation of amino acids within CFTR protein domains. I37 is located in the lasso motif (aa 1-69) of the CFTR protein. While the first 18 amino acids are poorly conserved, the remainder of this domain is highly conserved including the two lasso helices (Sabusap et al., 2021). I37 was conserved in 77% of the species investigated with only two conservative mutations to valine and leucine present in vertebrates. V37 was present in 48 species of ray-finned fish and one mammal (*Cricetulus griseus*), suggesting the mutation occurred after the divergence of ray-finned fish (Actinopterygii) from tetrapods and lobe-finned fish (Sarcopterygii) ~427 million years ago (Broughton et al., 2013). L37 was only present in the Florida manatee (*Trichechus manatus latirostris*). Statistical significance was determined using a two-tailed Wilcoxon rank sum test with Bonferroni correction. * adjusted p-value < 0.05, ** adjusted p-value < 0.01, *** adjusted p-value < 0.001, **** adjusted p-value < 0.0001. **(B)** Multiple sequence alignment of the lasso motif (aa 1-69) across representative vertebrate species. The conservation score represents the relative conservation of amino acid residues of CFTR and is divided into nine discrete grades from the most variable sites (grade 1; turquoise) to the most conserved sites (grade 9; maroon). The conservation score was calculated using the ConSurf Server with the multiple sequence alignment of CFTR from 230 vertebrate species (see **Table S5**).

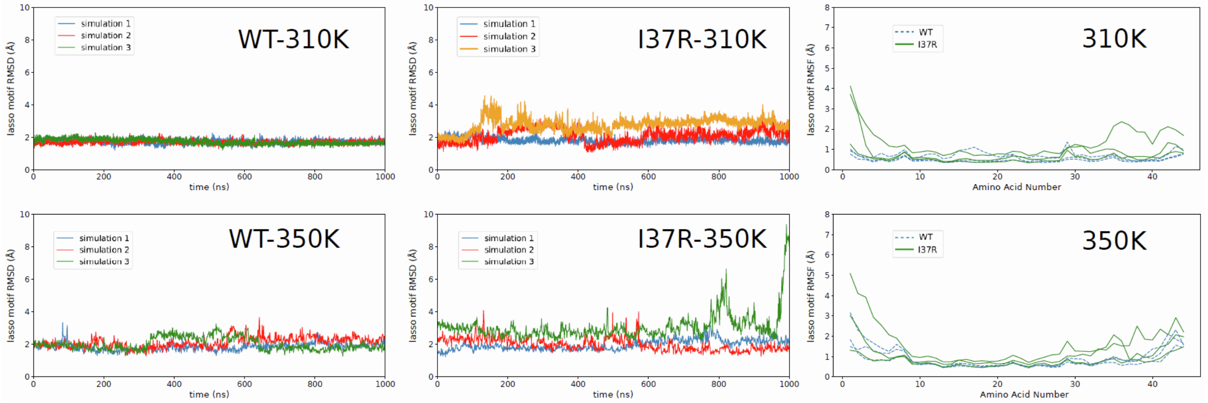

Figure S3. Comparison between the stability of the lasso motif in I37R-CFTR and WT-CFTR at 310K and 350K, related to Fig 4D. The lasso motif in the region around the I37R mutation was destabilised compared to the WT-CFTR lasso. At 310K, a stable conformation was only achieved in simulation 3, while the others required longer time to equilibrate. The data coloured orange denotes the simulation described in detail in the results section. The lasso in the WT-CFTR was stable at 350K.

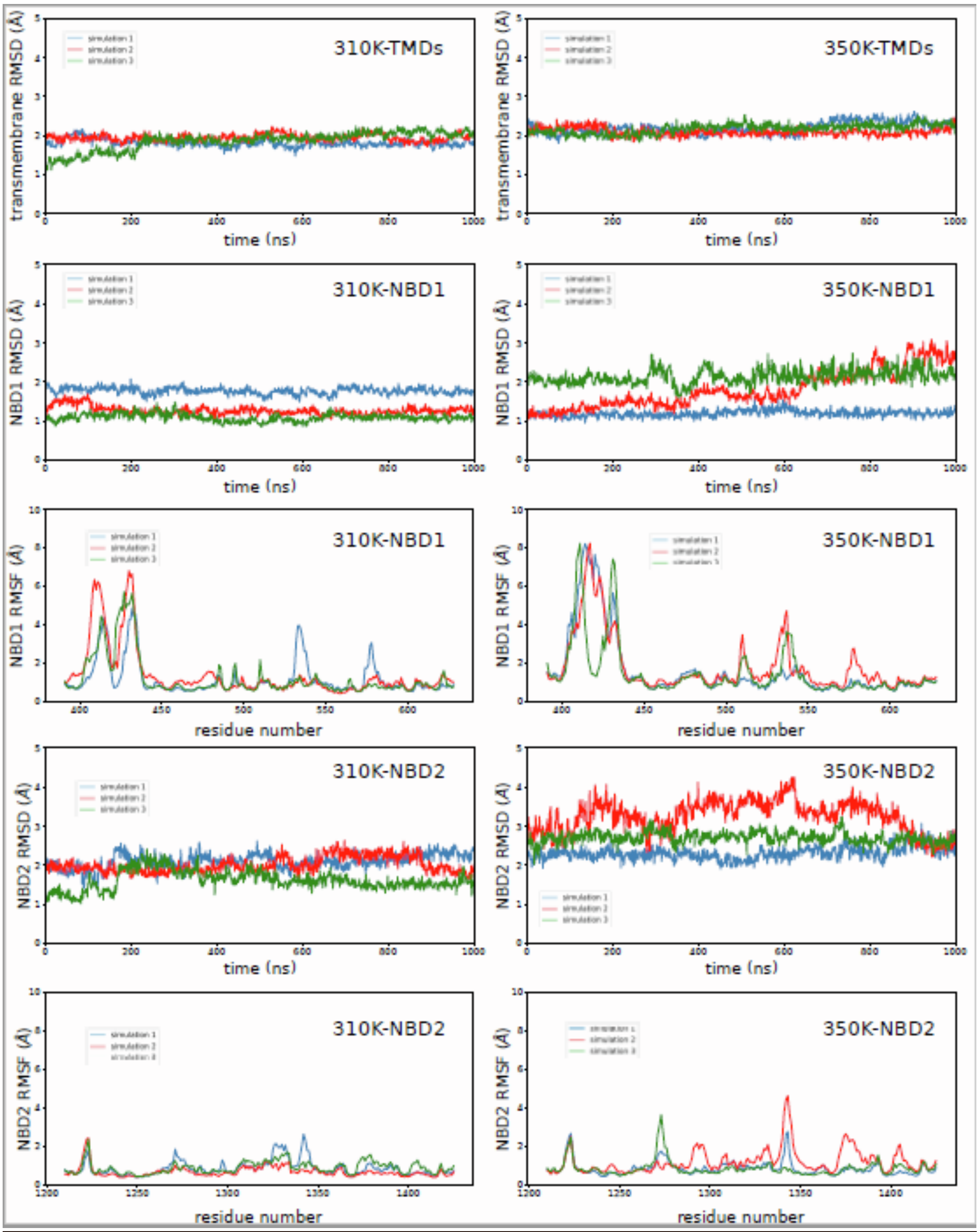

Figure S4. Comparison of the stability of different WT-CFTR domains at 310K and 350K, related to Fig 4D. (A) The transmembrane domains of WT-CFTR did not show significant conformational changes at the elevated temperature of 350K. **(B)** Small conformational changes in NBD1 were observed at 350K. The root-mean-square deviation (RMSD) for NBD1 was calculated using amino acids 391-628, excluding 401-440 as these amino acids compose the disordered Regulatory Insertion (RI). **(C)** The root-mean-square fluctuation (RMSF) profile for NBD1. The increase in RMSD at 350K was attributed to the already flexible sections. **(D)** NBD2 showed small conformational changes at 350K. The RMSD for NBD2 was calculated using amino acids 1226-1423. **(E)** The RMSF profile of the amino acids in NBD2 indicated that already flexible sections of NBD2 changed conformation at the elevated temperature while the rest of the domain remained stable.

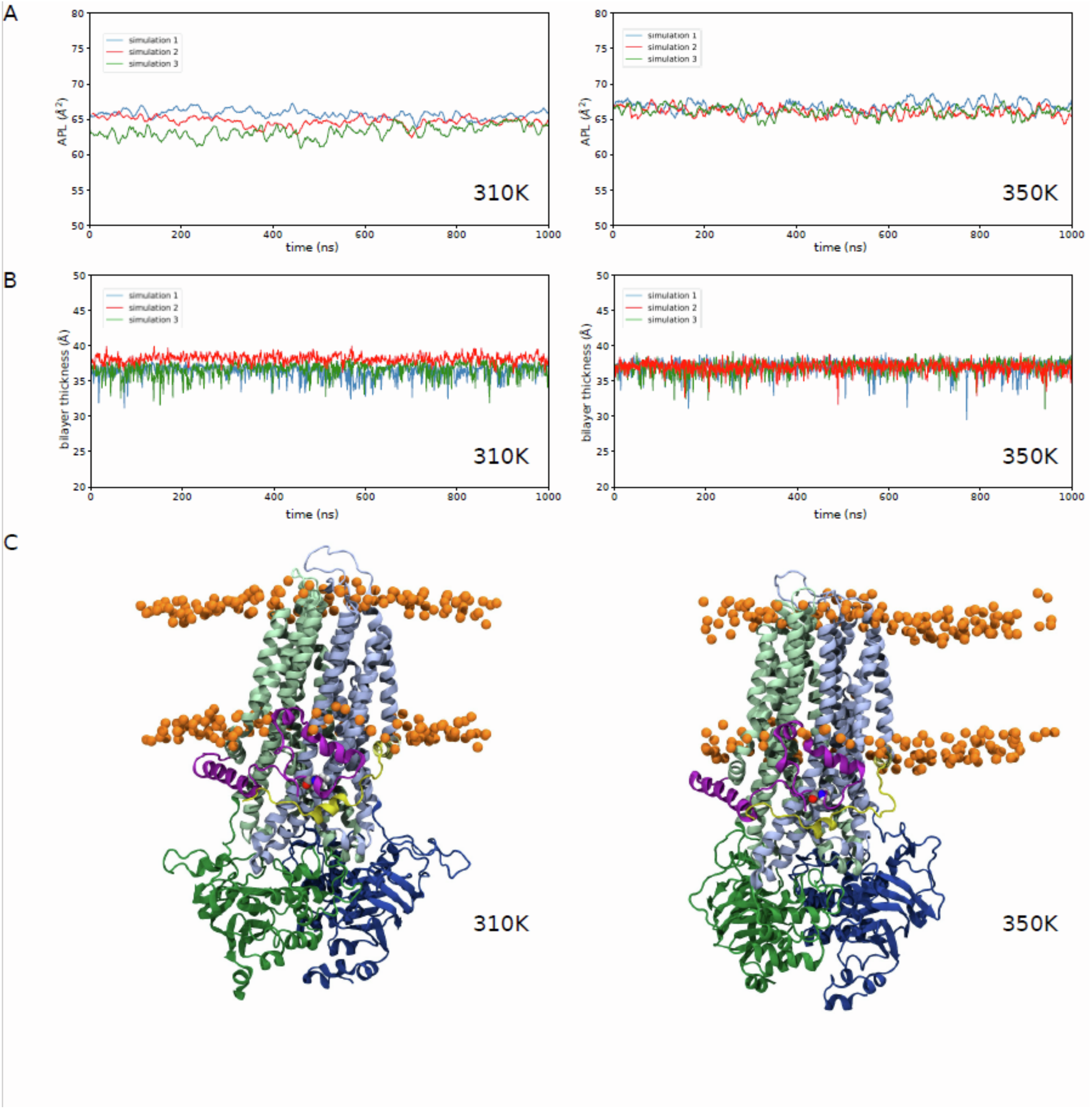

Figure S5. Comparison of the stability of the bilayer surrounding WT-CFTR at 310K and 350K, related to Fig 4D. (A) The area per lipid (APL) for the modelled 1-palmitoyl-2-oleoyl-sn-glycero-3-phosphocholine (POPC) bilayer was within expected tolerances at the elevated temperature of 350K. APL was plotted using a 10 ns moving average. **(B)** The thickness of the bilayer did not change significantly when simulated at 350K compared to 310K. **(C)** Visual inspection confirmed the stability of the bilayer at 350K (phosphate headgroups are depicted as orange spheres).

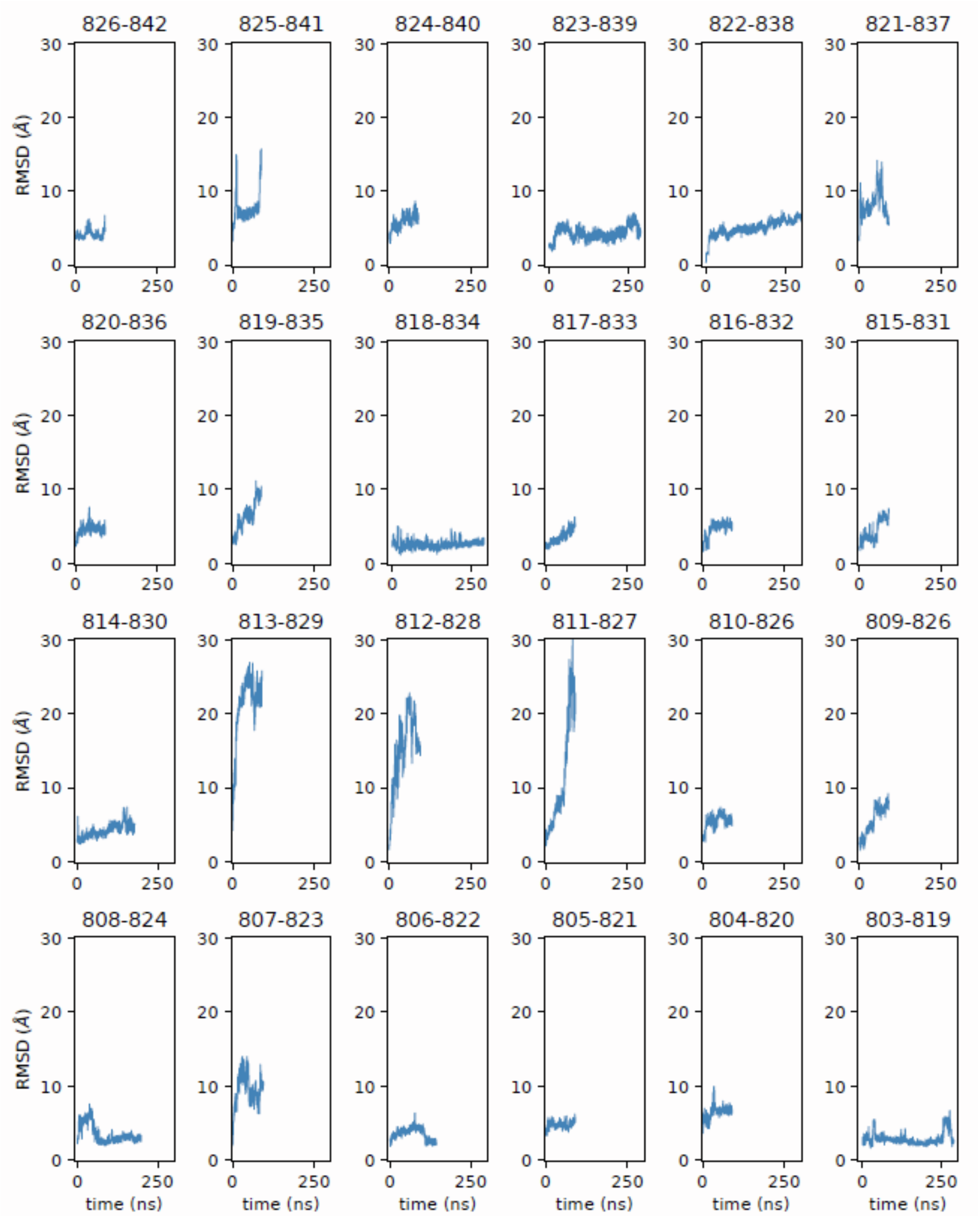

Figure S6. Root-mean-square deviation (RMSD) after molecular dynamics simulations of 24 models for the 17 unresolved residues of the R domain after the whole system was aligned to the transmembrane domains (TMDs) of the 6MSM structure, related to Fig 5B.

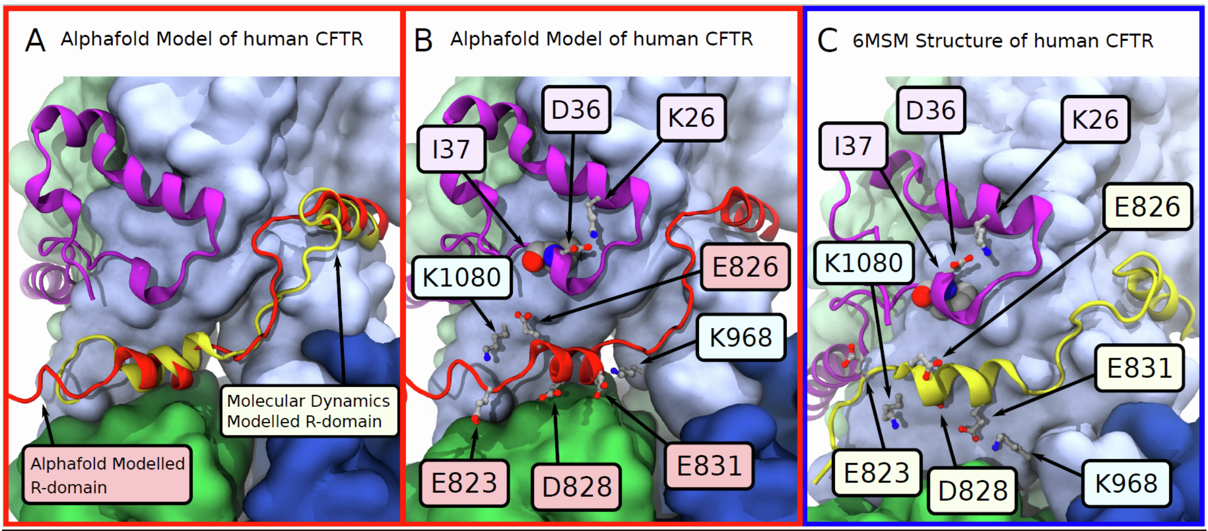

Figure S7. A comparison between the prediction of the structure of human CFTR by Alphafold2 and the modelled segment of the R domain, related to Fig 5B. Panels with red border indicate the background model in that panel is the Alphafold2 prediction, while the panel with blue border indicates the background model is that of 6MSM, the experimentally derived cryo-EM structure of human CFTR. The MD derived model of the R domain is coloured yellow while the Alphafold2 derived R domain is coloured red. **(A)** A closeup visualisation of the alignment between the Alphafold2 predicted R domain and the MD based prediction. This figure showed the agreement between the prediction of Alphafold2 and the MD model. The mouse, rat, human and zebrafish CFTR structures in the Alphafold2 database display the same tertiary structure, linking the helical R domain segment closest to the lasso motif, to the beginning of TMD2. This is consistent with the assignment of L818-F834 to the unknown segment of the 6MSM model using MD. **(B)** The placement of important charged sidechains in the structure predicted by Alphafold2. (hCFTR Alphafold2 model: https://alphafold.ebi.ac.uk/entry/P13569) **(C)** The placement of important charged sidechains in the 6MSM model, including the modelled helix.

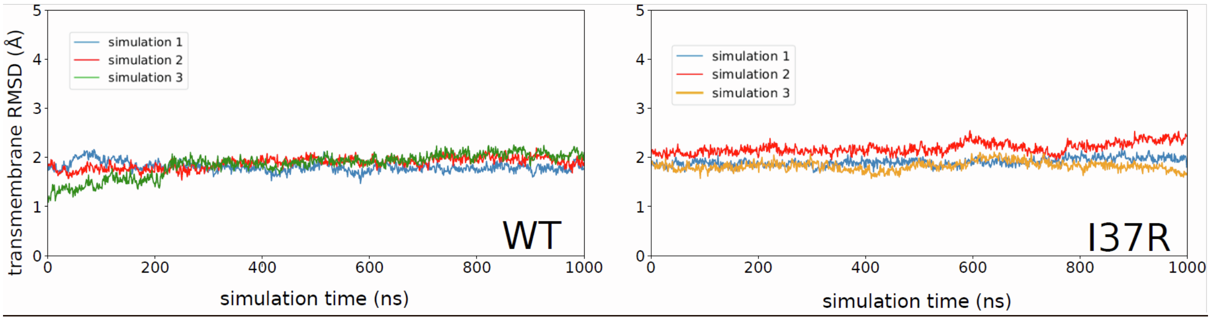

Figure S8. Comparison between the stability of the transmembrane domains in I37R-CFTR and WT-CFTR at 310K, related to Fig 4D. Throughout the microsecond simulations, the transmembrane domain of I37R-CFTR did not deviate significantly from the WT-CFTR indicating no allostery in the transmembrane domains of the I37R mutation. The data coloured orange denotes the simulation described in detail in the results section.

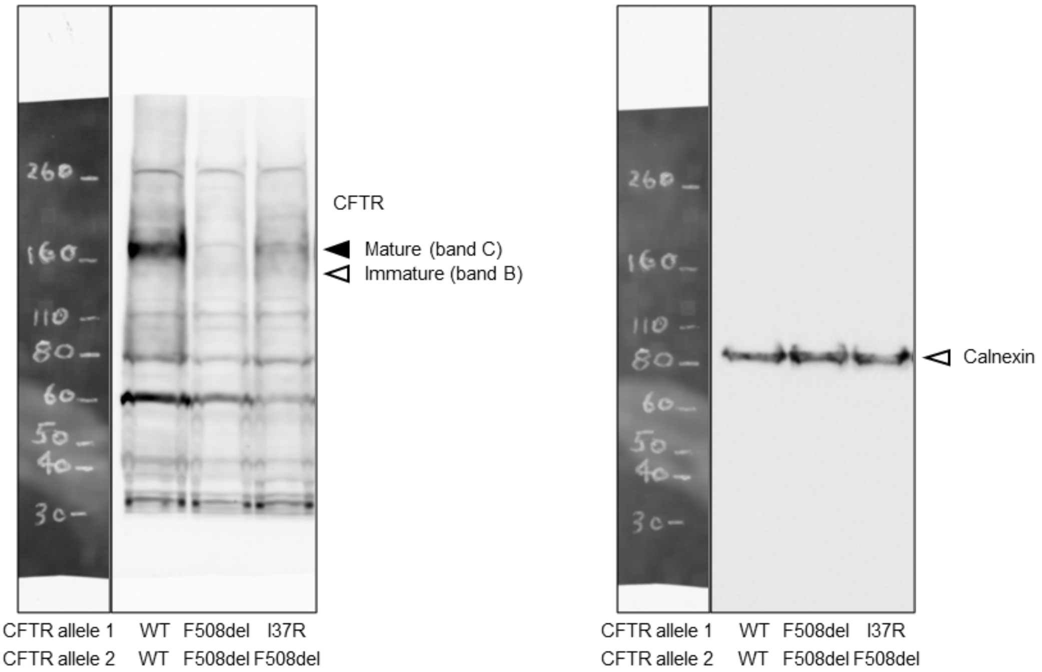

Figure S9. Original uncropped western blot of CFTR (A) and calnexin (B) in WT/WT, F508del/F508del and I37R/F508del intestinal organoids, related to Fig 1D.

Table S1. List of lasso motif mutations, related to Fig 1. Online excel-format table.

Table S2. Characteristics of study participants, related to STAR methods.

Participant ID	CFTR function/defect	*CFTR* genotype	Age (yr)	Sex	Exocrine pancreatic function status	Sweat Chloride (mmol/l)	ICM	Organoid FIS	2D organoid Isc
CF1	To be characterised	I37R/F508del	4.0	M	PS	81	Yes	Yes	Yes
CF2	Class II – Folding/maturation defect	F508del/F508del	4.1	F	PI	97	Yes	Yes	No
CF3		F508del/F508del	4.1	F	PI	92	Yes	Yes	No
CF4		F508del/F508del	0.8	F	PI	Nil	No	Yes	No
CF5		F508del/F508del	1.0	F	PI	Nil	Yes	Yes	No
CF6		F508del/F508del	9.0	M	PI	90	No	Yes	No
CF7		F508del/F508del	12.8	F	PI	76	No	No	Yes
CF8	Class III – Gating defect	G551D/F508del	0.4	F	PI	89	Yes	Yes	No
CF9		G551D/F508del	5.3	F	PI	94	No	Yes	No
WT1	Wild-Type function	WT/WT	10.9	M	NA	NA	No	No	Yes
WT2		WT/WT	5.7	M	NA	NA	Yes	No	No
WT3		WT/WT	16.2	M	NA	NA	Yes	No	No

FIS: forskolin-induced swelling; ICM: intestinal current measurement; Isc: short circuit current; PS: pancreatic sufficient; PI: pancreatic insufficient; NA: not applicable; Nil: Sweat chloride was not available.

Table S3. Electrophysiological parameters in rectal biopsies derived from CF and non-CF participants, related to Fig 1A.

**Electrophysiological parameters**	**WT/WT (n = 2)**	**F508del/F508del (n = 3)**	**G551D/F508del (n = 1)**	**I37R/F508del (n = 1)**
Basal resistance, R_{te} ($\Omega.cm^2$)	11.54 ± 1.22	15.86 ± 1.52	12.35 ± 2.15	19.53 ± 2.01
Amiloride-inhibited ENaC currents, ΔI_{sc} ($\mu A/cm^2$)	-27.05 ± 4.53	-16.17 ± 4.91	3.74 ± 0.6	-11.58 ± 6.53
cAMP-stimulated currents, ΔI_{sc} ($\mu A/cm^2$)	92.71 ± 9.79	11.68 ± 8.43	6.31 ± 0.58	45.76 ± 3.79
CCh-induced positive currents, ΔI_{sc} ($\mu A/cm^2$)	142.3 ± 27.92	NA	NA	NA
CCh-induced negative currents, ΔI_{sc} ($\mu A/cm^2$)	NA	-12.73 ± 2.73	-18.46 ± 4.78	-9.35 ± 2.53
cAMP+CCh-induced currents, ΔI_{sc} ($\mu A/cm^2$)	233.6 ± 34.47	1.20 ± 6.83	-12.14 ± 4.2	36.41 ± 2.43
CCh-induced biphasic peak currents, ΔI_{sc} ($\mu A/cm^2$)	NA	-2.28 ± 1.65	25.77 ± 2.16	15.78 ± 2.07
Bumetanide-inhibited currents, ΔI_{sc} ($\mu A/cm^2$)	-55.34 ± 5.82	9.24 ± 1.90	-15.15 ± 2.05	-22.43 ± 2.32

NA: not applicable

Table S4. Synergistic effect of dual potentiator combinations in FIS of I37R/F508del organoids, related to Fig 3A.

	**VX-770 + G1837**	**VX-770 + Gen**	**G1837 + Gen**
Sum of FIS of single potentiators	59.7+655.8=715.5	59.7+256.8=316.5	655.8+256.8=912.6
Synergy of dual potentiator combinations	774.1	672.9	1050.0
